# Quantifying the inundation impacts of earthquake-induced surface elevation change by hydrological and hydraulic modeling

**DOI:** 10.1038/s41598-021-83309-7

**Published:** 2021-02-19

**Authors:** Mao Ouyang, Yuka Ito, Tomochika Tokunaga

**Affiliations:** Department of Environment Systems, The University of Tokyo, Kashiwa, 277-8563 Japan

**Keywords:** Hydrology, Natural hazards

## Abstract

Current estimates of flood hazards are often based on the assumption that topography is static. When tectonic and/or anthropogenic processes change the land surface elevation, the spatial patterns of floods might also change. Here, we employ the hydrological and hydraulic modeling to simulate floods in the Kujukuri Plain, Japan, in the years 2004 and 2013, when two severe floods occurred. In between the two floods, land surface elevations were changed by the 2011 Tohoku-Oki earthquake. The effects of land surface elevation changes on inundation areas were quantified by changing input topographies. Our results showed that, without taking into account land surface elevation changes, around 10% of inundation areas were underestimated at the time of flood events in the year 2013. The spatial distribution of inundation locations varied with local topographical features, for example, the areas with backmarsh and valley fill deposits were sensitive to the extent of inundation by land surface elevation changes. The sub-watershed near the coastal shoreline having below-zero meter elevation areas showed that the earthquake-induced land surface elevation changes exacerbated an additional 22% inundation area. This study suggests that the inundation areas will increase in catchments suffering severe settlements, which highlights the necessity of taking into account the spatio-temporal changes of land surface elevations on the assessment of flood hazards.

## Introduction

Floods are one of the most common disasters, causing devastating catastrophes worldwide^1–3^. The intensity and severity of floods affect human society and its economic development^4–7^. Due to climate change, the floods are anticipated to increase in future^8–11^. Globally, 42% of the land shows an increase in the frequency of flood occurrence under the Representative Concentration Pathways (RCP) 8.5 (Ref.^12–14^). The local floods would even cause potentially negative repercussions with the propagation to the supply network and trade^1,15,16^. Therefore, understanding the physical and spatial characteristics of floods in a catchment scale would be of great benefit for developing mitigation measures and adaptation policies to reduce the negative influences.

Having fine resolution digital elevation models (DEM) is advantageous to investigate catchment scale flood events^17–20^. The flood model, which includes full integration of hydrological and hydraulic cycles, was suggested in constructing flood hazard maps^21^. The model can take into account the hydrodynamic features in the atmosphere, surface, and subsurface, which enables a realistic representation of the flood assessment^22–25^. Urbanization-induced land use and land cover change^26^, sea level rise^27^, and tidal effect^28^ can also be considered through the flood model. Land surface elevations are crucial in both overland and channel routing during heavy rainfalls, which can be adequately evaluated by the hydrological and hydraulic model.

Both natural and human activities can lead to land surface elevation changes, such as earthquake-induced tectonic movement^29,30^, sediment compaction^31–33^, coal mining^34^, sand mining^35^, and groundwater abstraction^36,37^. These temporal geomorphological processes were frequently occurred in coastal cities and caused significant impacts on flood hazards^38–41^.

**Figure 1:**
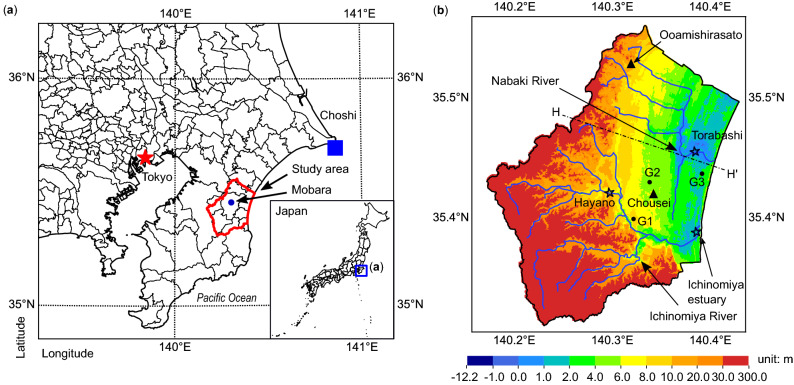
Location and topographic map of the study area. (**a**) The location of research site. Choshi is a tidal station, and the measured sea levels at Choshi were used as the coastal boundary conditions in the model. (**b**) The topographic map in the year 2013. Two river systems: Nabaki River and Ichinomiya River, flow through the study area. The Chosei station showed around 0.11 m subsidence, and the Oamishirasato station around 0.13 m subsidence due to the 2011 Tohoku earthquake^29^. Torabashi, Hayano and Ichinomiya Estuary are the river gauging stations, and the measured river water levels were used to calibrate model parameters (Fig. S3). G1, G2 and G3 are groundwater observation wells, and the measured groundwater levels were also used to calibrate the model parameters (Fig. S4). The modeled geological cross-section along the line H–H’ is shown in Fig. S2(c). (Figure generated using Matplotlib v. 2.2.3, http://www.matplotlib.org. Local basemaps from Geospatial Information Authority of Japan, Global Map Japan v. 2.2, http://www.gsi.go.jp, accessed 29 Jul 2020).

In this study, we employed the hydrological and hydraulic model to quantitatively evaluate the influence of land surface elevation changes on flood hazards in the Kujukuri Plain, Chiba Prefecture, Japan (Fig. 1a). The 2011 magnitude-9 Tohoku-Oki earthquake caused more than 0.1 m of subsidence in this area^29^. Specifically, the observed data showed around 0.11 m subsidence in the Chosei station, and 0.13 m in the Oamishirasato station. Figure 1b shows the topographic map in the year 2013. Two river systems—Nabaki River, with a catchment area of around 116.5 km^2^, and Ichinomiya River, with that of around 203.0 km^2^, flow through the study area. To examine the impacts of the 2011 earthquake-induced land surface elevation changes on the floods, the years 2004 and 2013 were chosen as the simulation periods because the typhoons Tokage and Wipha, respectively, hit the Kujukuri Plain^42,43^.

The hydrological and hydraulic model was applied in the research site by employing the coupled MIKE SHE^44^ and MIKE HYDRO River^45^ software to evaluate the inundation areas (see “Methods” section for more details). The simulated scenarios are listed in Table 1. The sensitive parameters, e.g., Manning’s coefficients and hydraulic conductivities, were calibrated by comparing the simulation results with the observations during the years 2018 and 2019 (Scenario Cal in Table 1). The flood events in the years 2004 and 2013 were simulated as cases 04A and 13A (the numbers represent the year of input topography, and the letter “A” represents the actual case), and compared with the surveyed inundation area. Comparisons between simulated and observed river water levels, groundwater levels, and the extents of floods, revealed that the developed flood model can reasonably well reproduce the hydrological processes in the area. Subsequently, the input topographies of the cases 04A, 13A, were changed to the topographies of 2013 (13P, “P” represents the parametric case) and 2004 (04P), respectively, while keeping the others unchanged, to quantify the effects of land surface elevation changes on flood features. Our results indicated that, without considering the land surface elevation change, the inundation areas were underestimated at around 10%.

**Table 1:** The simulated scenarios for calibration, validation, and quantification of the hydrological and hydraulic model. Cal means the simulation for calibration. The numbers in the scenarios column represent the year of input topography, and the letter “A” is for the actual simulations and “P” is for the parametric simulations.

Scenarios	Purpose	Topography	Simulation period	Examination duration	Typhoon	TP*	MP**
Cal***	Calibration	2013	2018.01–2019.10	2018.12 and 2019.04–2019.07	No	604	17.5
04A	Actual	2004	2004.01–2004.12	2004.10.08–2004.10.09	Tokage	209	49.0
13A	Actual	2013	2013.01–2013.12	2013.10.15–2013.10.16	Wipha	272	42.0
04P	Parametric	2004	2013.01–2013.12	2013.10.15–2013.10.16	Wipha	272	42.0
13P	Parametric	2013	2004.01–2004.12	2004.10.08–2004.10.09	Tokage	209	49.0

*Total precipitation during the examination period (mm).

**Hourly maximum precipitation during the examination period (mm).

***There was no typhoon during the examination duration.

## Results

### Comparison with observations

**Figure 2:**
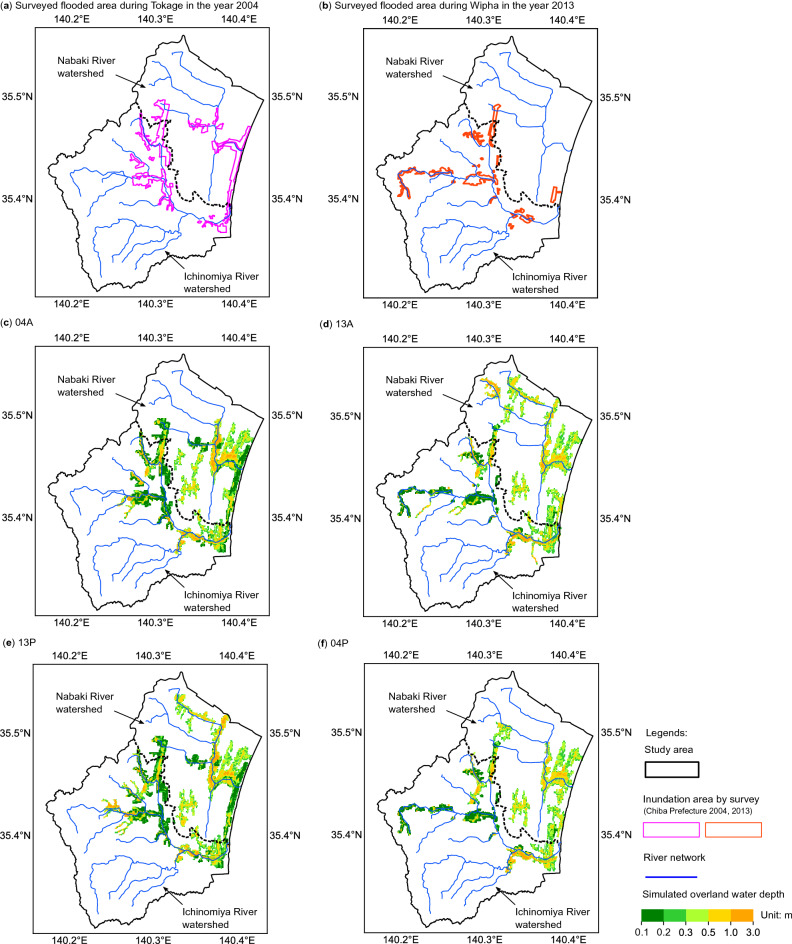
Observed and simulated inundation areas in both Nabaki and Ichinomiya River watersheds for four scenarios. The regions surrounded by magenta lines in (**a**) were the reported flooded areas in the year 2004 (Ref.^42^), and those surrounded by the orange lines in (**b**) were the surveyed flooded areas mainly in the Ichinomiya River watershed at the flood event in the year 2013 (Ref.^43^). (**c**) 04A and (**d**) 13A were employed to compare the calculated flooded areas with the observed ones. (**e**) 13P and (**f**) 04P were conducted to quantify the influence of land surface elevation changes on the spatial features of the inundation areas. The boundary of river watersheds is based on Dutta et al.^18^. (Figure generated using Matplotlib v. 2.2.3, http://www.matplotlib.org).

The simulated inundation areas of the four scenarios (Table 1) are presented in Fig. 2, together with the observations. The regions surrounded by magenta lines in Fig. 2a were the reported flooded areas by Chiba Prefecture at the time of flood event in the year 2004 (Ref.^42^), and those surrounded by orange lines in Fig. 2b are those at the flood event in the year 2013 (Ref.^43^). The field investigation in the year 2013 was mainly conducted in the Ichinomiya River watershed and not thoroughly in the Nabaki River watershed; therefore, the following quantitative discussions were only focused on the Ichinomiya River watershed. Table 2 shows the surveyed and calculated extents of the inundation areas. A comparison of the field investigated (Fig. 2a,b) and calculated (Fig. 2c,d) inundation areas indicated that the simulation could reproduce around 98% of the surveyed flooded area for both cases. For the simulated inundation areas in the Ichinomiya River watershed, approximately 80% of the simulated flooded areas were reported to be flooded in both the years 2004 and 2013.

**Table 2:** Comparison of observed and simulated flood extents.

Actual scenarios	Surveyed area^42,43^	Calculated area	Parametric scenarios	Calculated area
04A	15.0 km^2^	18.6 km^2^	13P	20.4 km^2^
13A	14.3 km^2^	17.1 km^2^	04P	14.0 km^2^

The numerical results suggested a potential overestimation of the extents in the inundation areas, which might be resulted from the spatial resolution of overland flow modeling. The resolution of original DEM is 1 m with the accuracy in elevation to be 0.03 m. The river cross-sectional profiles were obtained from official report, 1 m resolution DEM, and site investigation, thus, the levees and embankments were accounted for in the channel flow simulation by MIKE HYDRO River. However, the overland flow was simulated in the 50 m size cells by MIKE SHE, upscaled from the 1 m resolution DEM, due to the compromise between the computational power and accuracy. Even though this caused an enlargement of depression areas, which then led to an overestimation of the inundation areas compared to the observed flood extents, the fact that the numerical modeling can reproduce most of the reported inundation areas indicated that the current model could yield reasonable quantification of land surface elevation changes on the inundation impacts.

### Quantification on inundation areas

When comparing the inundation areas of 04A with 13P (Fig. 2c,e), and 13A with 04P (Fig. 2d,f), for the same examination durations but different topographies, the inundation maps demonstrated that the changes of land surface elevations would alter the total inundation areas and spatial patterns of flood hazards.

**Figure 3:**
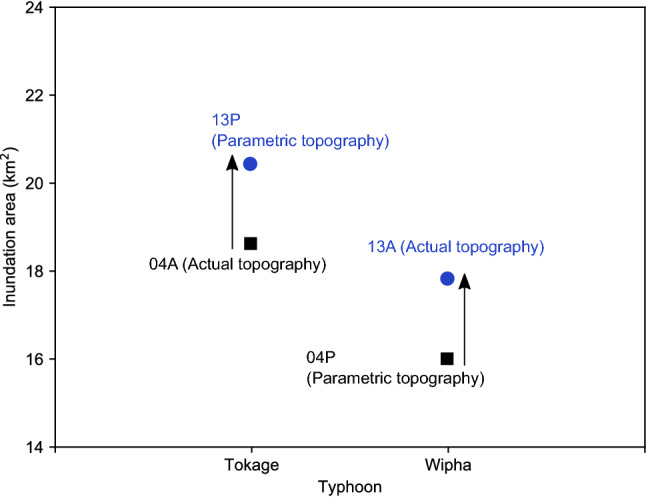
Quantification of the inundation areas considering the effects of land surface elevation changes in the Ichinomiya River watershed. 04A and 04P are the cases with the topographies before the earthquake, while 13P and 13A are those after the earthquake, which represents the results considering the influence of earthquake-induced land surface elevations.

A summary of the total inundation areas is shown in Fig. 3. For the typhoon Tokage, the calculated result with the topography of the year 2004 showed around 18.6 km^2^ of flooded area in the Ichinomiya River watershed, whereas, that with the topography of the year 2013 around 20.4 km^2^, indicating additional 10% were calculated to be possibly inundated due to land surface elevation change. A similar increase of inundation areas with typhoon Wipha was also noted in Fig. 3, which illustrated that the land surface elevation changes could exaggerate the inundation areas in the study site during the examination period.

### Spatial distribution of inundation areas

**Figure 4:**
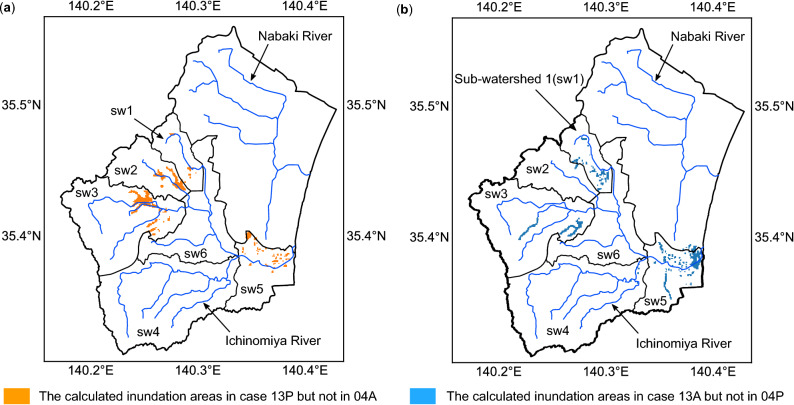
Comparison of the simulated inundation areas at the time of flood events in the year 2013 and 2004, respectively. (**a**) The difference of the calculated flooded areas between 04A and 13P, i.e., the area flooded by 13P but not by 04A. (**b**) The difference of the calculated flooded areas between 13A and 04P, i.e., the area flooded by 13A but not by 04P. (Figure generated using Matplotlib v. 2.2.3, http://www.matplotlib.org).

The difference of the calculated flooded areas between 04A and 13P, i.e., the area flooded by 13P but not by 04A, is shown in Fig. 4a. A similar comparison between 13A and 04P was shown in Fig. 4b. For both flood events in 2004 and 2013, the sub-watersheds 4 and 6 showed small inundation areas, and the majority of inundation locations with different input topographies were almost the same. In the flood plain area, at the time of flood event in 2004, sub-watersheds 1, 2, 3, and 5 showed differences in the calculated flood patterns. Sub-watersheds 1, 3, and 5 showed differences at the 2013 flood event.

**Figure 5:**
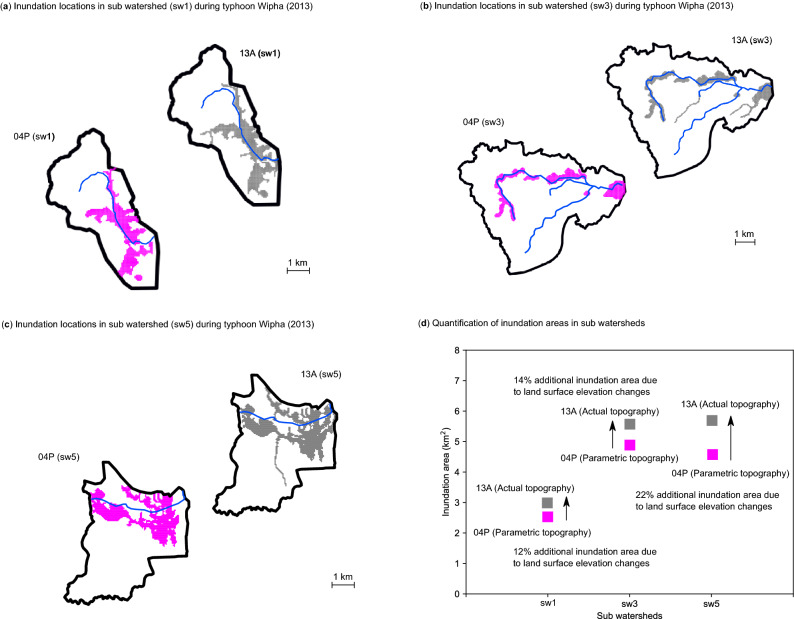
Detailed examination of the inundation areas in sub-watersheds during typhoon Wipha in 2013. (**a**) Inundation locations in sub-watershed 1. (**b**) Inundation locations in sub-watershed 3. (**c**) Inundation locations in sub-watershed 5. (**d**) Quantification of inundation areas in the sub-watersheds (Figure generated using Matplotlib v. 2.2.3, http://www.matplotlib.org).

To quantify the spatial distribution of flood hazard affected by earthquake-induced surface elevation change, the details of inundation locations in sub-watersheds 1, 3, and 5 at the 2013 flood event in cases 13A and 04P are presented in Fig. 5. The simulated inundation areas of cases 13A and 04P in subwatersheds 1, 3, and 5 are summarized in Table 3. Regarding sub-watershed 1, an additional 12% were calculated to be possibly flooded by the land surface elevation changes during the typhoon Wipha. In sub-watershed 3, around 14% of inundation areas would be underestimated if the topography in the year 2004 was applied for the simulation. The sub-watershed 5 showed that more than 4.6 km^2^ of the land was suffered from flooding for both 13A and 04P cases. An additional 22% of the lands were calculated to be flooded by the land surface elevation changes.

**Table 3:** Summary of the calculated inundation areas during 2013 typhoon Wipha in sub-watersheds 1, 3, and 5.

Scenarios	Simulated inundation areas in sub-watershed (km^2^)		
	sw1	sw3	sw5
13A	2.9	5.6	5.6
04P	2.6	4.9	4.6

Based on the 1 m resolution DEMs obtained by the Light Detection and Ranging (Lidar) and used in this study, we obtained the subsidence map in between the years 2004 and 2013 in the study area. The whole area showed subsidence due to the 2011 Tohoku-Oki earthquake. Since the results and discussions were mainly focused on the Ichinomiya River watershed, the subsidence of the Ichinomiya River watershed was presented in Fig. S1(a). Figure S1(b) showed the difference of the calculated flooded areas between 13A and 04P, i.e., the area flooded by 13A but not by 04P. The difference of the simulated inundation areas by hydrological and hydraulic model (Fig. S1(b)) noted that the Ichinomiya River watershed would not be totally inundated although the whole area showed subsidence (Fig. S1(a)). More specifically, the inundation areas might not be exacerbated in some locations where severe subsidence was presented, suggesting that the locations where earthquake caused surface elevation change would not be always correlated with the locations where additional inundation was occurred.

## Discussion

**Figure 6:**
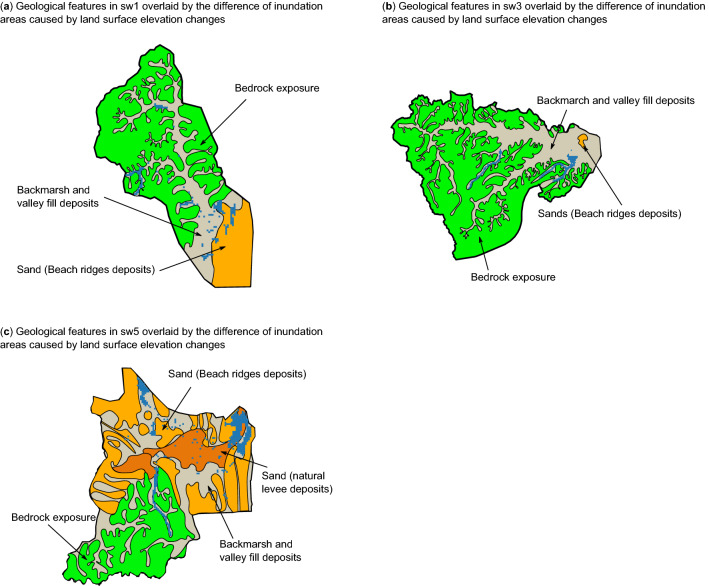
Simplified geologic map of the sub-watersheds (**a**) sw1, (**b**) sw3, and (**c**) sw5, overlaid by the difference of inundation areas during typhoon Wipha in the year 2013. (Figure generated using Matplotlib v. 2.2.3, http://www.matplotlib.org. Local geological maps from Geological Survey of Japan, http://www.gsj.jp, assessed 29 Jul 2020).

Our results showed that the surface elevation changes would affect the total inundation areas and the spatial patterns of floods in the coastal basin at the time of floods. Because the characteristics of geology were considered to affect the extent of the flood hazards, the simplified geologic map of sub-watersheds 1, 3, and 5 (Refs.^46,47^), overlaid by the difference of inundation areas, are presented in Fig. 6. The additional inundation areas calculated to be accounted for by land surface elevation changes in sub-watersheds 1 (Fig. 6a) and 3 (Fig. 6b) indicated that areas containing backmarsh and valley fill deposits and enclosed by terraces or beach ridges were sensitive to floods by elevation changes. For example, in sub-watershed 3, the majority of the difference of inundation areas was found in the valley areas (Fig. 6b). The valleys in sub-watersheds 1, 2, and 3 only have small outlets for the discharge of river water flow, which possibly affects the inundation features during heavy rainfall periods. This influence might be exaggerated by the surface elevation changes, resulting in some additional inundation areas in sub-watersheds 1 and 3 at the flood event in the year 2013. The surface elevations in sub-watershed 5 (Fig. 6c) were very low, and some areas were even below zero meters, which makes it quite vulnerable to floods. The simulation results showed that the elevation changes would exacerbate the inundation areas in sub-watershed 5 by 22% in the 2013 flood events. Our study demonstrated that the spatial impacts of land surface elevation changes on the inundation areas vary with locations, which can be impacted by the geological characteristics, and to obtain a comprehensive picture of the flood hazard map, the hydrological and hydraulic flood modeling can be useful to provide more detailed information.

Comparison of inundation areas in between the years 2004 and 2013 (04A, 13A in Fig. S2(a) and (b)) revealed that the inundation patterns were not always constant and would be affected by the precipitation patterns, which were reported to be an important factor controlling the flood hazards^22,48^. The spatial distributions of precipitation during the typhoons Tokage and Wipha, respectively, were obtained from the composite data of radar and Automated Meteorological Data Acquisition System (AMeDAS)^49,50^ (Fig. S2(c) and (d)). The spatial resolution of the precipitation data in the year 2004 was 2.5 km mesh, and that in the year 2013 was 1.0 km mesh, respectively. To compare these two cases, precipitations of the years 2004 and 2013 were remapped to the same resolution with 0.05 km using a simple nearest-neighbor remapping algorithm. Observation of the precipitation at the time of the 2013 flood event (Fig. S2(d)) revealed that the sub-watershed 3 received a large amount of rainfall, which might have corresponded to the flood hazards along the river in sw3 (Fig. S2(b)). During the typhoon Tokage in 2004, the intensive rainfall in sub-watershed 3 was not recorded (Fig. S2(c)), thus, the inundation was not observed (Fig. S2(a)). Interestingly, the sub-watershed 5 received less rainfall during the typhoon Wipha (2013) than during the typhoon Tokage (2004). However, the confluence zones suffered severer flood hazard in the 2013 flood event (Fig. S2(b)) than that in the 2004 flood event (Fig. S2(a)), as indicated in blue square boxes. This might be responsible for the river flow features, i.e., sub-watershed 4 is situated in the upstream of the Ichinomiya River which received a large amount of rainfall in 2013 and flowed to the downstream, and it resulted in severe flood hazards in the confluence zones located in the intersection of sw4, sw5, and sw6 (Fig. S2(a) and (b)).

The inundation areas were expanded due to land surface elevation changes in the study site during the examination period. The framework presented here to account for the land surface elevation changes on flood hazards is transferable to other cities and can be used to assess the flood risks and inform policy decisions affecting environmental activities. New approaches and models are still needed for better understanding and representation of the dynamic features of floods^51,52^. It was reported that employing the new elevation data could triple estimate the coastal flood vulnerability due to sea level rise^39^, and here we presented that earthquake-induced land surface elevation changes exacerbate the inundation areas due to heavy rainfall. The results of this study suggested that for large-scale flood hazard assessment, the spatio-temporal changes of elevations are necessary to be taken into account in preparing the model parameters. The topography considering the spatio-temporal changes within the designated period should be used as the input DEM to assess the flood hazard. The study site, the Kujukuri Plain, has also been suffering from continuous land surface elevation changes, i.e., around 0.01 m per year subsidence at maximum, possibly due to groundwater exploitation^37^. The next step would tackle the effect of groundwater abstraction-induced land subsidence on the changes in the possible flood patterns and the assessment of future floods.

## Methods

### Research flow

Both earthquake and flood are natural hazards which would cause devastating catastrophes. Land surface elevation changes caused by earthquake has been reported in some catchments^29,30^. In this study, we aim to quantify the flood hazard impacts of earthquake-induced surface elevation change. The focus of this research was not to discuss the effects of uncertain topography on the flood hazard^53–56^, but to quantify the inundation areas in the catchments where certain earthquake-induced surface elevation change was observed. To reach this target, the hydrological and hydraulic model was employed to map the flood inundation areas^57^. The calibration, validation against measured data, and uncertainty analysis against the most affecting factors^58,59^, were conducted to ensure that the model was appropriate to evaluate the flood hazard. Our study presented an approach to link one hazard, earthquake-induced surface elevation change, with another, flood, and demonstrated that the spatio-temporal changes of topography was necessary to be considered in flood model.

### Model principle

We coupled MIKE SHE^44^ and MIKE HYDRO River^45^ to evaluate the inundation impacts of land surface elevation changes in the Kujukuri Plain. Following processes were included in the model: the spatially distributed precipitation, evaporation and transpiration; 1D Saint Venant’s equation for the river channel flow; 2D shallow-water equation for the overland flow based on the kinematic routing; 1D Richards’ equation for the unsaturated zone; 3D groundwater flow. The model has been widely applied for both academic types of research and engineering management, focusing on the water management^60^, climate effects on hydrology^61^, water quality^62^ and eco-hydrology^63^ etc.

### Model development

The total area of the model domain is 370.5 km^2^ with a spatial cell size of 50 × 50 m in MIKE SHE. The chosen resolution produced 148,187 cells in horizontal plane within the model domain. The thickness of each cell is 0.1 m for the simulation of subsurface flow.

The river network was digitized in the MIKE HYDRO River from the raster data. The river cross-sectional profiles were modeled based on the official reports^64^, 1 m resolution DEM, and site investigation, which suggested that the river levees and embankments were considered in the hydrological and hydraulic model. The high resolution DEMs were measured by Lidar with targeting vertical error of 0.05 m in our case. The leveling data in the study area were also obtained, and were compared with the Lidar data. The standard deviation of the Lidar data to the leveling data was 0.03 m, which suggested that the Lidar data showed a smaller vertical error than the targeting one. Figure S3 presented the histogram of the land surface elevation changes of the input data obtained from the 1 m resolution Lidar DEMs of the years 2004 and 2013 in the Ichinomiya River watershed. The downstream boundaries of the rivers were set to follow the sea levels observed at the Choshi station. Manning’s channel roughness coefficient was calibrated and listed in Table S1.

Due to the limitation of computational power, for the simulations of overland flow and subsurface flow in MIKE SHE, the input topography was remapped to 50 m resolution by inverse distance weighting through 1 m resolution DEM.

The land use land cover data were obtained from Chen et al.^36^. They constructed the land use land cover data from the Landsat-5 TM image, which was divided into eight classes, i.e., building estate, cropland, forest, grassland, paddy field, sands, urban, and water (Fig. S4(a)). The mean reproduction through the comparison between the band classified results and aerial photography was around 86% for each class^36^. The leaf area index (LAI) and root zone depth were obtained through the Ref.^17^, and presented in Table S1.

The subsurface formation was divided into three layers, i.e., 2-m shallow soil layer, the Holocene unconsolidated sand unit, and low-permeable Plio-Pleistocene Kazusa Group in descending order^65^, which was illustrated in Fig. S4(b). The hydraulic conductivities were treated as calibration parameters, and were shown in Table S1. The boundary conditions of saturated groundwater flow at the coast were assigned as hydrostatic pressure equal to seal level, and other boundaries were assigned as no flow.

The distribution of surface soil profile was obtained from the Chiba Prefecture official reports (Refs.^46,47^), with the spatial resolution of 50 m. Seven categories of soils were applied to this study area^36^, i.e., gley soil, immature soil, peat, gley lowland soil, brown lowland soil, brown forest soil, and andosols soil (Fig. S4(c)). The parameters for calculating unsaturated flow were obtained from the previous research^17^, and were listed in Table S1.

Composite precipitation data of Radar and Automated Meteorological Data Acquisition System (AMeDAS)^49,50^ were the data source for the spatially distributed precipitation. The spatial resolution of the precipitation data in the year 2004 was 2.5 km, and that in the year 2013 was 1.0 km. A simple nearest-neighbor remapping algorithm was used to allow the comparison in the same resolution of 0.05 km. The accuracy of the Radar/Raingauge-Analyzed precipitation was 96% (Refs.^49,50^). Actual evapotranspiration was calculated from the potential evaporation and soil moisture in the root zone by Kristensen and Jensen method^66^.

### Parametric study

After developing the model, the hydrological process was calibrated through the comparison of simulated and observed river water levels (Fig. S5); and the groundwater levels (Figs. S6 and S7), by bootstrapping^67^. The detailed comparison of simulated and observed river water levels at the Torabashi, Hayano and Ichinomiya Estuary stations (Fig. S5(b), (d), and (f)) showed good agreements, suggesting that the model and parameter settings were reasonable to reproduce the hydrodynamic processes. The calibration ranges of the parameters within 3-standard-deviations from the observations are listed in Table S1.

**Figure 7:**
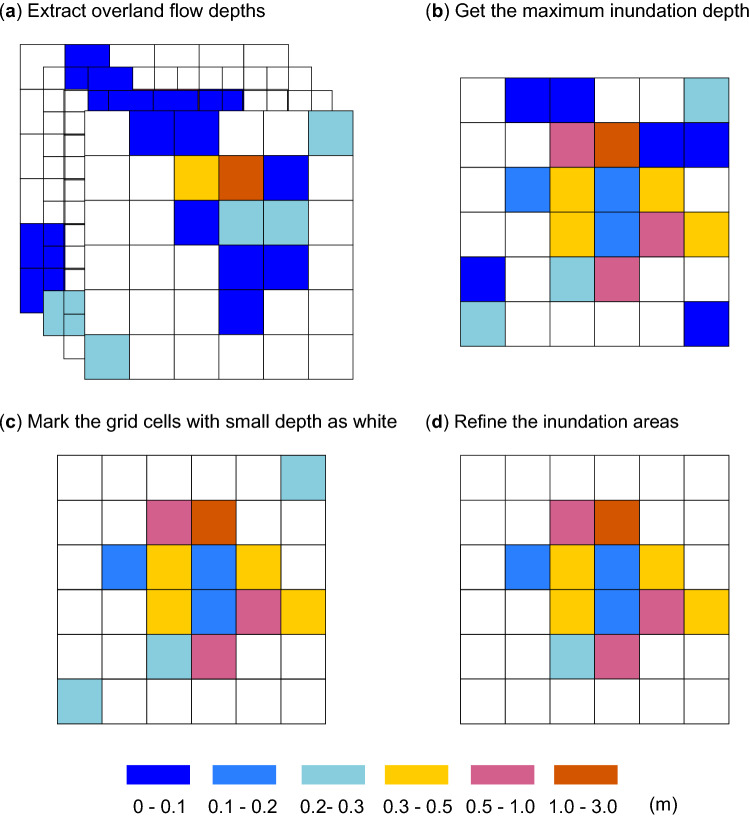
Method of obtaining the inundation areas from the calculated results. (**a**) Collect the calculated overland flow depths with a one-hour interval during the examination periods. (**b**) Compare and obtain the maximum inundation depth in each cell. (**c**) Mark the cells with the small inundation depth (0.1 m) as white. (**d**) Refine the inundation areas to ignore the areas where the number of connected cells is smaller than 10.

To ensure the appropriateness of the parameters used, and to examine the characteristics of floods, the typhoon Tokage which attacked the study area from 8 to 9 Oct 2004, and the typhoon Wipha from 15 to 16 Oct 2013, were selected to further validate the models (scenarios 04A and 13A). The good reproducibility of the flooded areas (see “Inundation map projection” section, Fig. 7) by comparing the simulated results with the actual flooded areas (Fig. 2; Table 2) was achieved, and the parameters were considered to be appropriate in this study. In order to investigate the necessity of considering the earthquake-induced elevation changes in the hydrological and hydraulic modeling, the input topographies and the patterns of precipitations were interchanged. Accordingly, the inundation areas and spatial distribution of flooded locations can be quantified and discussed. Although the simulation of flood in 2004 using the topography of 2013 (13P) would not contribute to the practical implications, two simulations considering the change of surface elevation could increase the persuasiveness of our results.

### Inundation map projection

Figure 7 demonstrates the approaches of obtaining inundation area maps in this study. The computed depths of overland flow during the examination periods were collected first with a one-hour interval during the examination periods. Through the comparison of water depths at the same locations, the maximum inundation depth map was obtained^22^. Then, the grid cells with very small inundation depth were marked as white (0.1 m in this study) based on the assumption that the very small water depth might generally not cause disasters^12,28^. Finally, the inundation map was refined by ignoring the regions where the connected number of grids are smaller than 10 in this study, which might represent the small rivers and lakes, and was hard to be surveyed in the field.

### Parameter sensitivity

Most of the parameters in the developed dynamic flood model were derived from the related previous studies^17,36,65^, while the Manning’s roughness coefficient of rivers were empirically determined^19^. To answer the question if a small change in the Manning’s roughness coefficient of rivers makes large enough change in flood extent without the need to account for the modified topography, the sensitivity of the flood model to Manning’s channel roughness coefficient was studied by keeping all but the Manning’s channel roughness coefficient fixed in the scenarios 04A and 13P, respectively. According to Arcement and Schneider^68^, the Manning’s channel roughness coefficients were estimated to be varied from 0.017 to 0.025 for the channels with fine sands, therefore, the cases that changed the Manning’s channel roughness coefficient by -20% (n = 0.016) and + 20% (n = 0.024) were conducted, and were named M-20 and M + 20, respectively. The obtained flood extents were then compared with the reference models (04A and 13P). The calculated inundation areas in the Ichinomiya River watershed were illustrated in Fig. S8. The flood patterns were rarely altered by the change of Manning’s channel roughness coefficient within the range of the changes in this study. Figure S9 quantified the sensitivity of Manning’s channel roughness coefficient to the inundation areas. Results revealed that the simulated inundation area became larger (smaller) when the Manning’s channel roughness coefficient was larger (smaller). Twenty percent change of Manning’s channel roughness coefficient caused around 2% change of inundation areas. This is because flooding from river channels happens more easily when the roughness coefficient is larger^19^. According to the 1D Saint Venant’s equation, the river flow velocity becomes slower with larger roughness coefficient, so that water tends to be inundated to the surrounding floodplains rather than being discharged to downstream side.

For the same Manning’s channel roughness coefficients, the simulated inundation areas with the input topography of the year 2013 (simulations based on 13P) were always larger than those with input topography of the year 2004 (simulations based on 04A). The change of topography caused around 10% change of the simulated inundation areas.

Besides the Manning’s channel roughness coefficient, the input precipitation was suggested to be another affecting source of uncertainty according to global sensitivity analysis in flood inundation model^57,59^. Japan Meteorological Agency^50^ reported that the precipitation obtained from AMeDAS showed an error of 4%. Thus, the sensitivity analysis was conducted by changing the amount of distributed precipitation with − 4% and + 4% in each grid cell, and was named P − 4 and P + 4, respectively. The inundation areas with the sensitivity of input precipitation were compared with reference models (04P and 13A), as shown in Fig. S10. The results indicated that the calculated inundation areas were not strongly affected by the change of ± 4% input precipitation. However, the change of topography resulted in around 10% change of the simulated inundation areas. This suggested that the exacerbation of flood extent due to dynamic topography was significant enough to be distinguishable from the changes in the flood extents caused by the uncertainty of Manning’s channel roughness coefficient and the input precipitation.

### Limitation

It should be noted that our calculated inundation area is an estimation based on the current state of precision, and the quantities in the results should be interpreted with caution because of several methodological challenges. The DEM, precipitation, and land use land cover patterns have uncertainty in the representation of flood events because of their spatial resolutions. The parameters of the model could also have uncertainty in computing the hydrological processes. The other limitation is that during the substantial rainfall period, the inundation flow was reported to be strongest near the levees, and erosion features were frequently found^69^. The eroded deposits vary their features corresponding to the types, extents, intensity of floods and surrounding environments. These replenished sediments would change the geomorphological situation of the rivers and affect flow directions^70^, which might change the inundation characteristics during extreme weather. An improved understanding of the feedback effects, the effects of floods on land surface elevation changes, would be useful to fully understand the compound hazards between floods and the land surface elevation changes^71^.

## Supplementary Information

Supplementary Information.

## Data Availability

The spatial distributed precipitations and sea levels are obtained from the Japan Meteorology Agency^49,50^. The high-resolution digital elevation data are measured with Lidar by Kokusai Kogyo Corporation. Earthquake-induced land surface elevation data are available from the Geospatial Information Authority of Japan^29,30^. Land cover data are constructed from the Landsat-5 TM images^36,37^. The soil profiles are obtained from the National Land Agency of soil map in Chiba Prefecture. The river network was digitized from the raster data, and the cross-sections are obtained from official reports^64^ and site investigations. The elevations of groundwater levels are measured by the Keiyo Natural Gas Association. The river water levels, surveyed inundation areas during the flood events are provided by the government of Chiba Prefecture^42,43,64^. All data needed to evaluate the conclusions in this contribution are presented in the paper.
