# Supplementary material for: Quantifying the inundation impacts of earthquake-induced surface elevation change by hydrological and hydraulic modeling
Source: Sci Rep. 2021 Feb 19;11:4269. doi: 10.1038/s41598-021-83309-7 (PMC7895827; doi:10.1038/s41598-021-83309-7)
Supplement: Supplementary file 1 — Supplementary Information. [file 41598_2021_83309_MOESM1_ESM.docx]

**Supplementary materials**

Quantifying the inundation impacts of earthquake-induced surface elevation change by hydrological and hydraulic modeling

Mao Ouyang^1*^, Yuka Ito^1^, and Tomochika Tokunaga^1^

^1^ Department of Environment Systems, The University of Tokyo, Kashiwa, 277-8563, Japan

^*^ einooumo@hotmail.com

Table S1. The calibrated parameters in the hydrological and hydraulic model. The references noted in the “Component” column are the ones from which the values were taken. The calibration ranges of the parameters within 3-standard-devation are also included in the “Value” column.

| Component | Parameter | Distribution | Value |
| --- | --- | --- | --- |
| Evapotranspiration  (Dutta et al.^17^) | Leaf area index (LAI) (-) | Building estate | 10 |
|  |  | Cropland | 200 |
|  |  | Forest | 400 |
|  |  | Grassland | 300 |
|  |  | Paddy | 250 |
|  |  | Sands | 15 |
|  |  | Urban | 10 |
|  |  | Water | 5 |
|  | Root depth (mm) | Building estate | 0.2 |
|  |  | Cropland | 20 |
|  |  | Forest | 50 |
|  |  | Grassland | 30 |
|  |  | Paddy | 25 |
|  |  | Sands | 5 |
|  |  | Urban | 0.2 |
|  |  | Water | 0.5 |
| Channel flow | Manning’s *n* (s/ [m^1/3^]) | Constant | 0.016 – 0.024 |
| Overland flow  (Dutta et al.^17^) | Surface roughness coefficient *n* (s/ [m^1/3^]) | Building estate | 0.009 – 0.017 |
|  |  | Cropland | 0.024 – 0.045 |
|  |  | Forest | 0.120 – 0.225 |
|  |  | Grassland | 0.040 – 0.075 |
|  |  | Paddy | 0.028 – 0.053 |
|  |  | Sands | 0.024 – 0.045 |
|  |  | Urban | 0.010 – 0.018 |
|  |  | Water | 0.014 – 0.027 |
|  |  |  |  |
| Unsaturated subsurface flow  (Chen et al.^36^) | Saturated moisture content (-) | Gley soil | 0.30 |
|  |  | Immature soil | 0.28 |
|  |  | Peat soil | 0.28 |
|  |  | Grey lowland soil | 0.30 |
|  |  | Brown lowland soil | 0.30 |
|  |  | Brown forest soil | 0.30 |
|  |  | Andosols soil | 0.40 |
|  | Saturated hydraulic conductivity (m/s) | Gley soil | 1e-7 – 5e-6 |
|  |  | Immature soil | 1e-6 – 5e-4 |
|  |  | Peat soil | 1e-8 – 5e-7 |
|  |  | Grey lowland soil | 1e-6 – 5e-5 |
|  |  | Brown lowland soil | 1e-6 – 5e-5 |
|  |  | Brown forest soil | 1e-7 – 5e-6 |
|  |  | Andosols soil | 1e-6 – 5e-5 |
|  | Residual moisture content (-) | Uniform | 0.01 |
|  | α (related to the inverse of the air entry suction) (-) | Uniform | 0.067 |
|  | *n* (measure of pore-size distribution) (-) | Uniform | 1.446 |
| Saturated subsurface flow | Hydraulic conductivity (m/s) | Holocene sand unit | 4e-4 – 6e-4 |
|  |  | Plio-Pleistocene Kazusa Group | 1e-10 – 5e-9 |


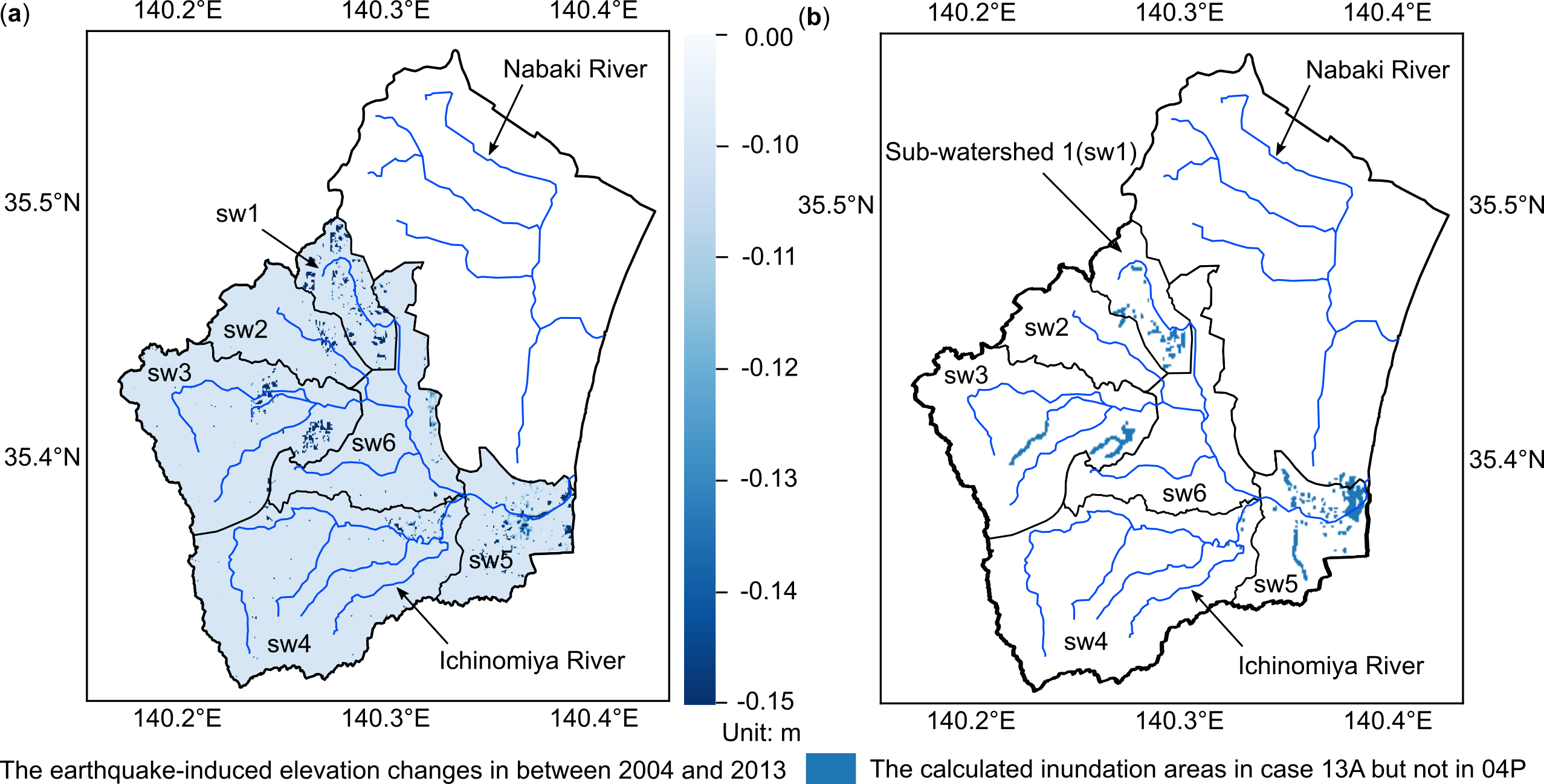


Figure S1. (a) The earthquake-induced land surface elevation changes in between 2004 and 2013 in the Ichinomiya River watershed. Note that negative values indicate subsidence. (b) The difference of calculated inundation areas between 13A and 04P, i.e., the area flooded by 13A but not by 04P. (Figure generated using Matplotlib v. 2.2.3, [www.matplotlib.org](http://www.matplotlib.org/).)


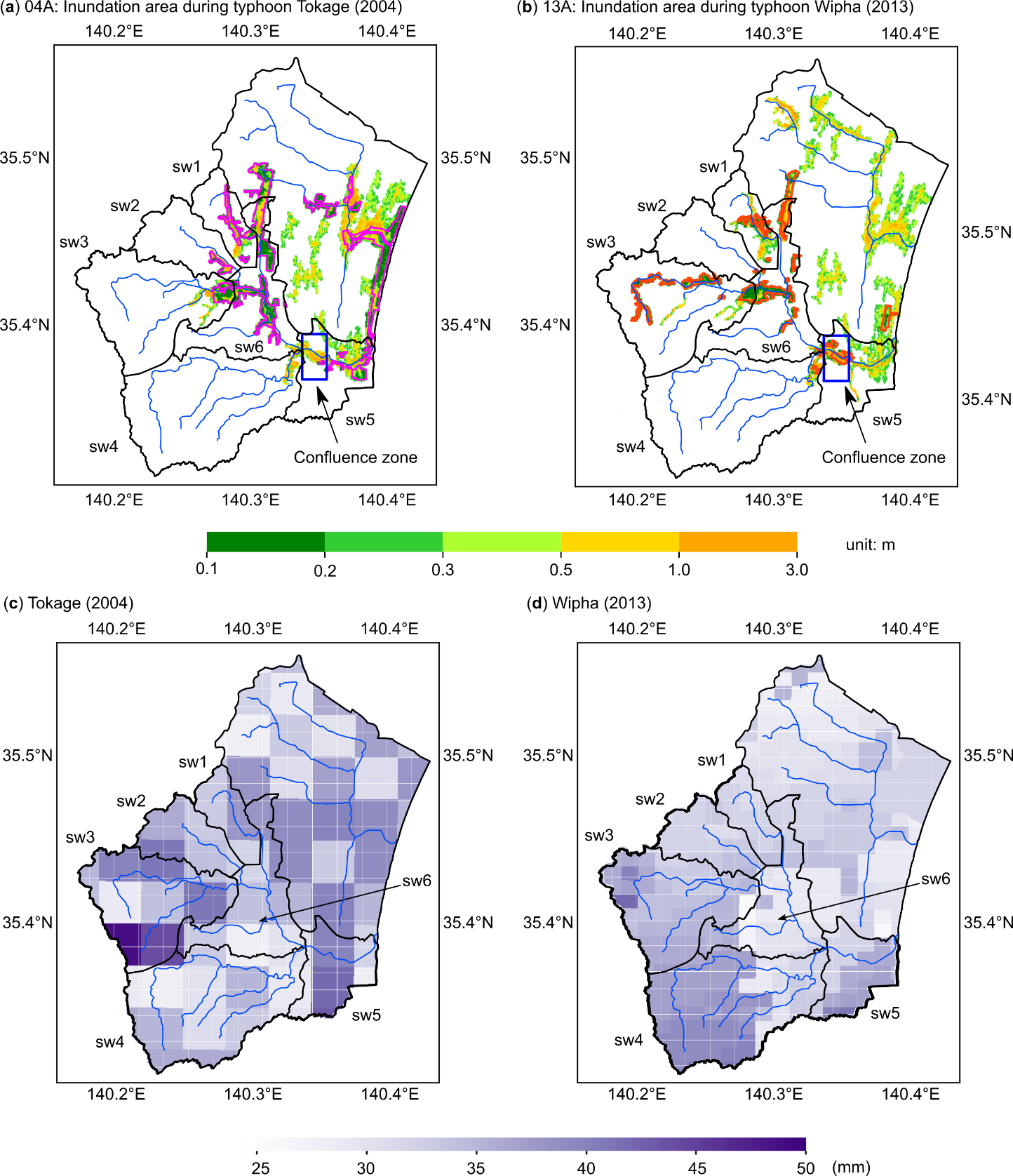


Figure S2. Inundation areas of the actual scenarios and spatial distribution of hourly maximum precipitation during the typhoon Tokage 2004 (ref^48^) and Wipha 2013 (ref^49^) in sub-watersheds. (a) Inundation areas of actual case 04A. (b) Inundation areas of actual case 13A. The survey was not thoroughly conducted in the Nabaki River watershed in the year 2013. (c) Spatial distribution of precipitation during typhoon Tokage in the year 2004 (ref^42^). (d) Spatial distribution of precipitation during typhoon Wipha in the year 2013 (ref^43^). (Figure generated using Matplotlib v. 2.2.3, [www.matplotlib.org](http://www.matplotlib.org/).)


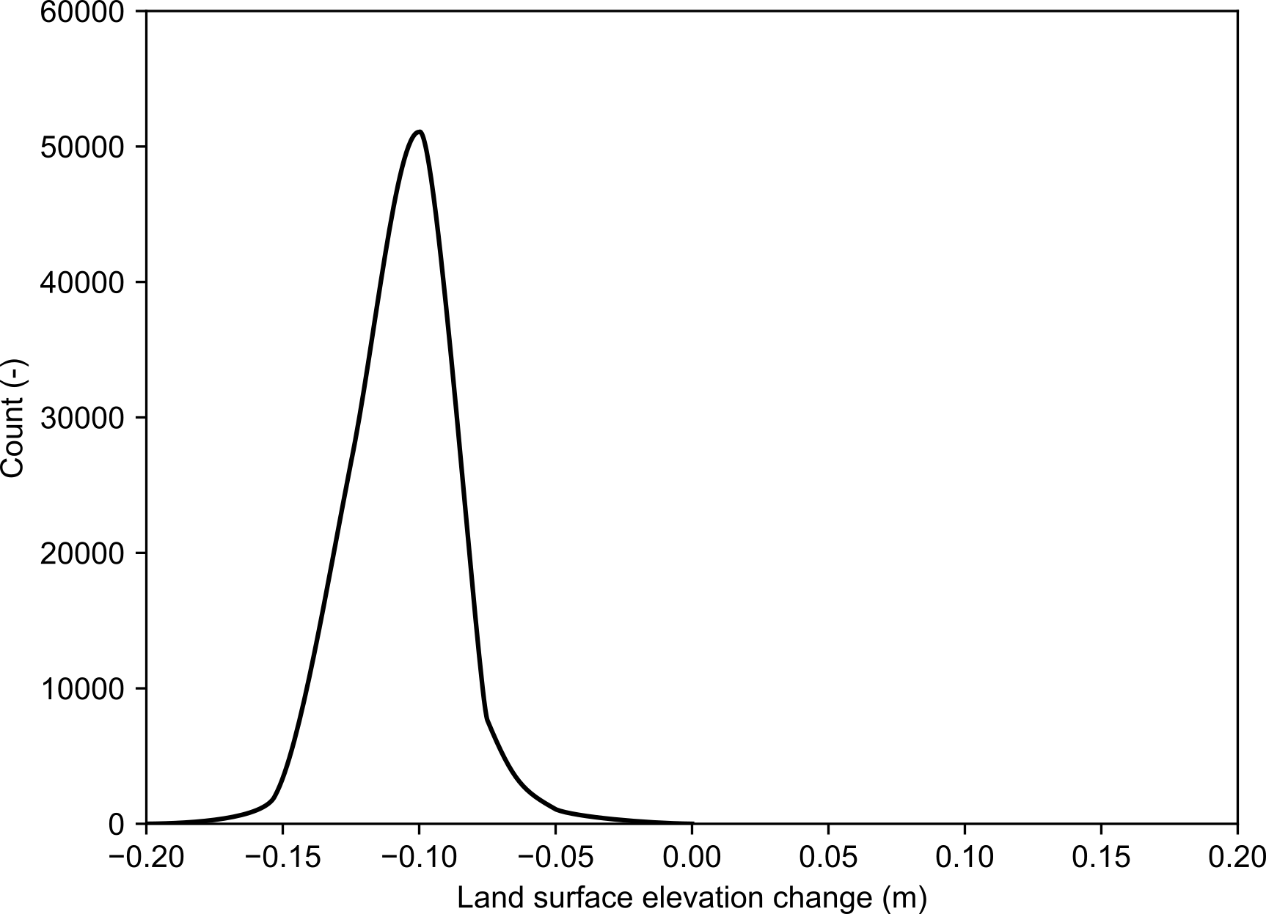


Figure S3.The histogram of the land surface elevation changes in between the years 2004 and 2013 in the Ichinomiya River watershed.

`
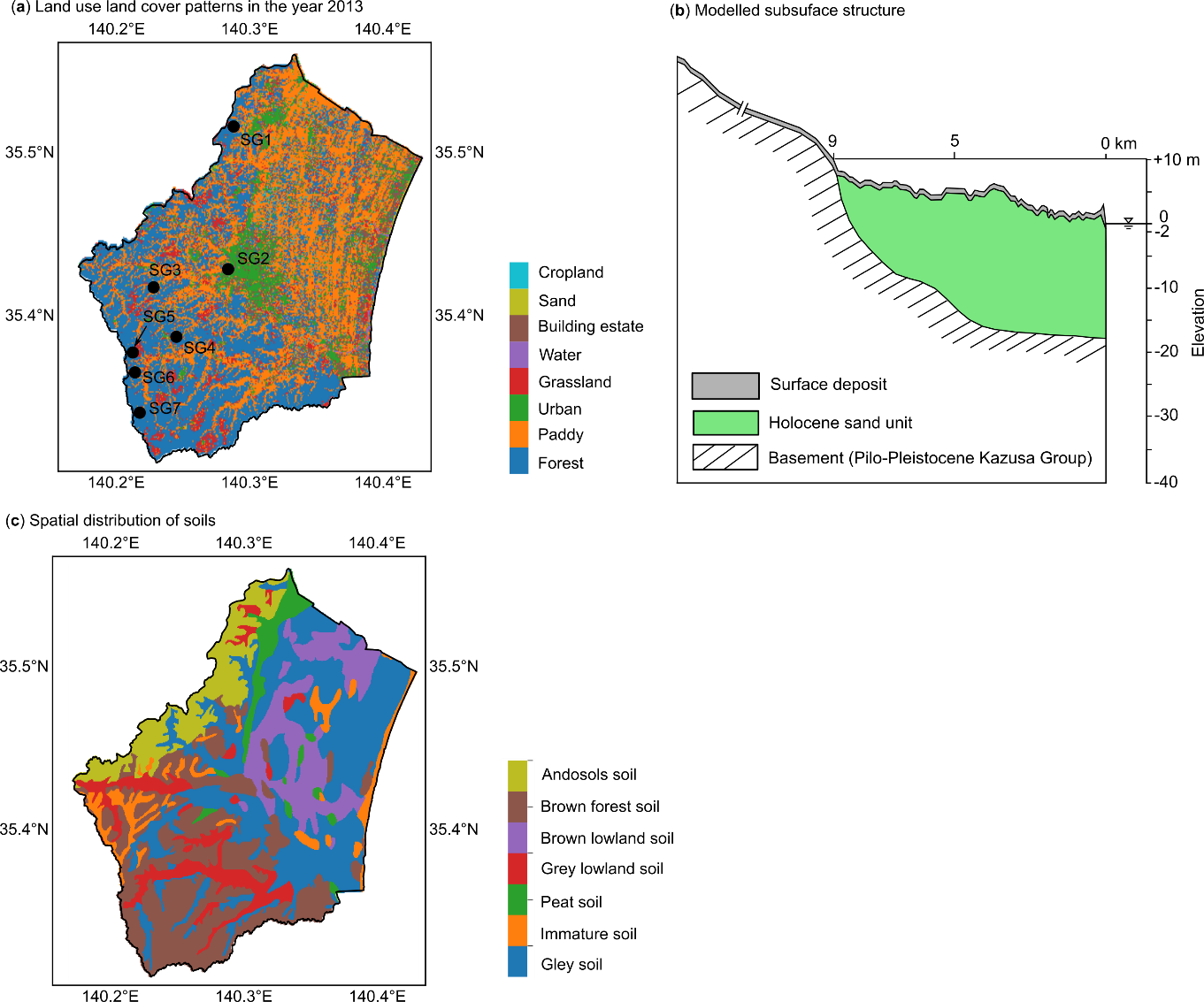


Figure S4. Surface and subsurface models in this study. (a) The land use land cover patterns in the year 2013, which controls the surface roughness of overland flow, leaf area index, and root zone in the model. SG1 – SG7 are the locations for measuring the groundwater discharge elevations. (b) The simplified geological cross section in this model. Location of the cross section H-H’ is shown in Fig. 1(b). (c) Spatial distribution of soils in this model. (Figure generated using Matplotlib v. 2.2.3, [www.matplotlib.org](http://www.matplotlib.org/).)


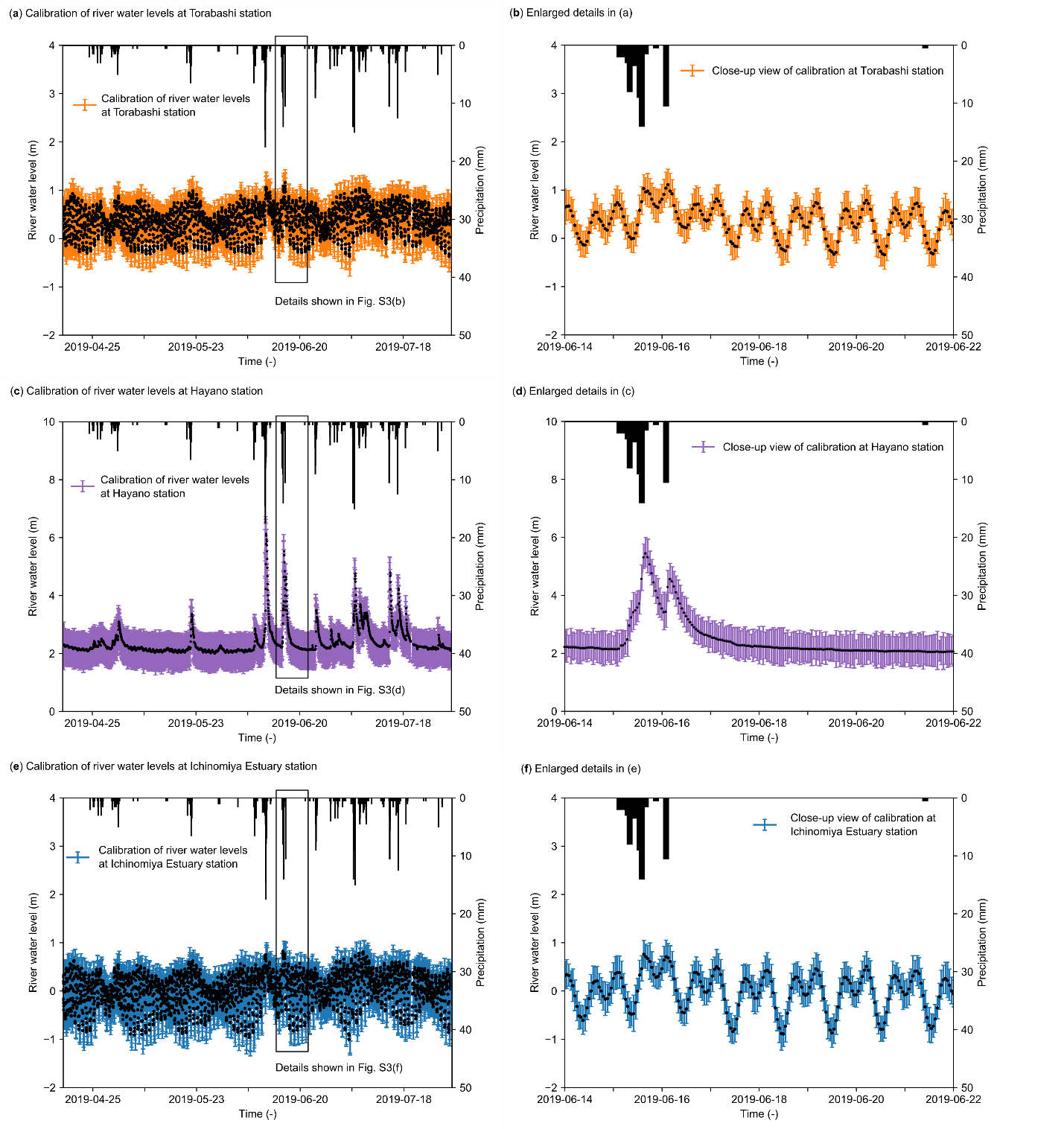


Figure S5. Calibration of river water levels at the Torabashi, Hayano, and Ichinomiya Estuary stations. Locations of the stations are shown in Fig. 1(b). Black dots in each figure indicate the measured river water levels. Bars in each figure indicate hourly precipitation at the Mobara station (ref Fig. 1(a)). The error bars show 3-standard-deviation uncertainties and were obtained through bootstrapping. (a) Calibration of river water levels at Torabashi station. (b) Close-up view of the period indicated in (a). (c) Calibration of river water levels at Hayano station. (d) Close-up view of the period indicated in (c). (e) Calibration of river water levels at Ichinomiya Estuary station. (f) Close-up view of the period indicated in (e).


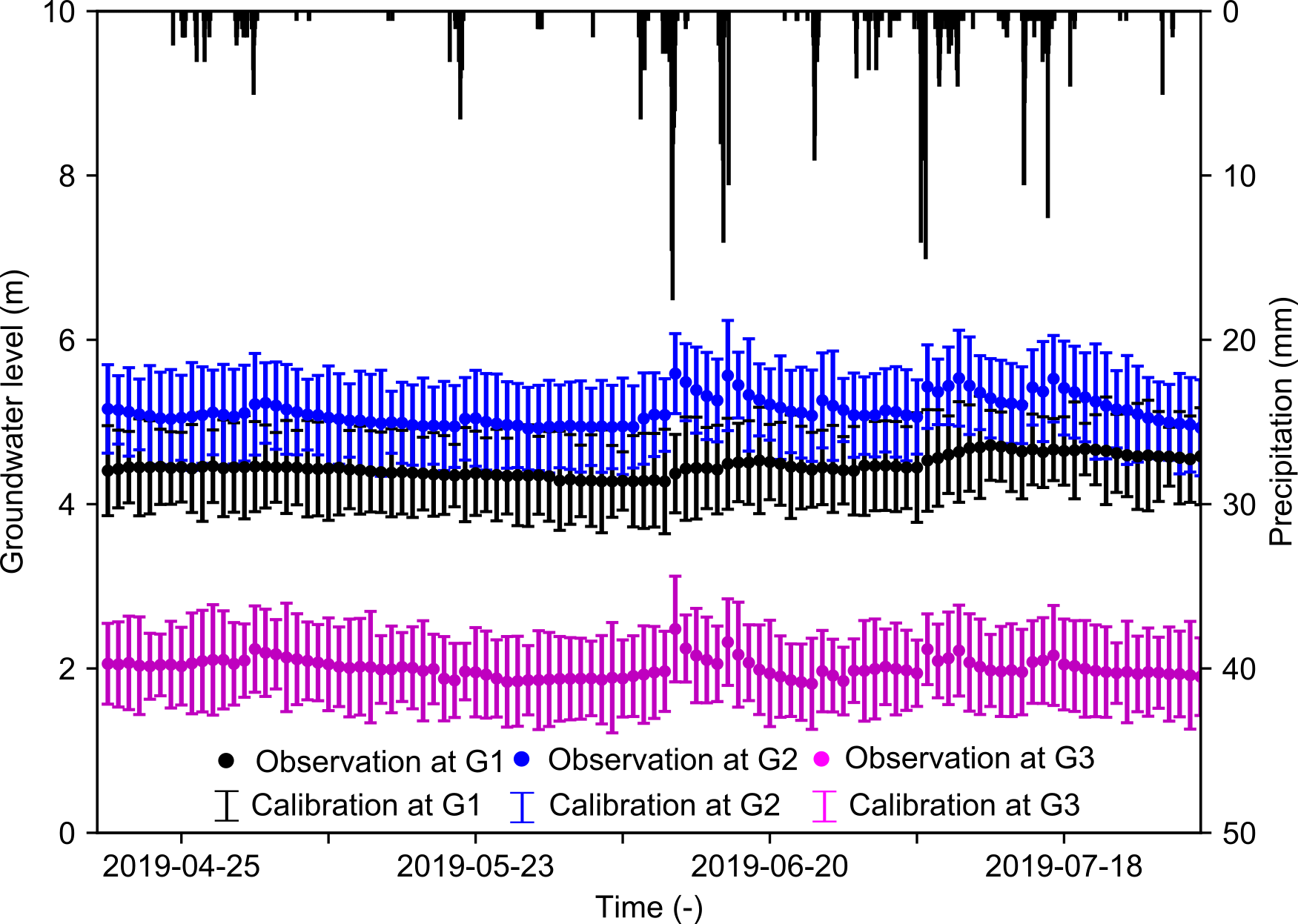


Figure S6. Calibration of the temporal groundwater levels at the G1, G2, and G3. The locations of the observation stations are shown in Fig. 1(b). Bars in the figure indicate hourly precipitation at the Mobara station (ref Fig. 1(a)). The error bars show 3-standard-deviation uncertainties and were obtained through bootstrapping.


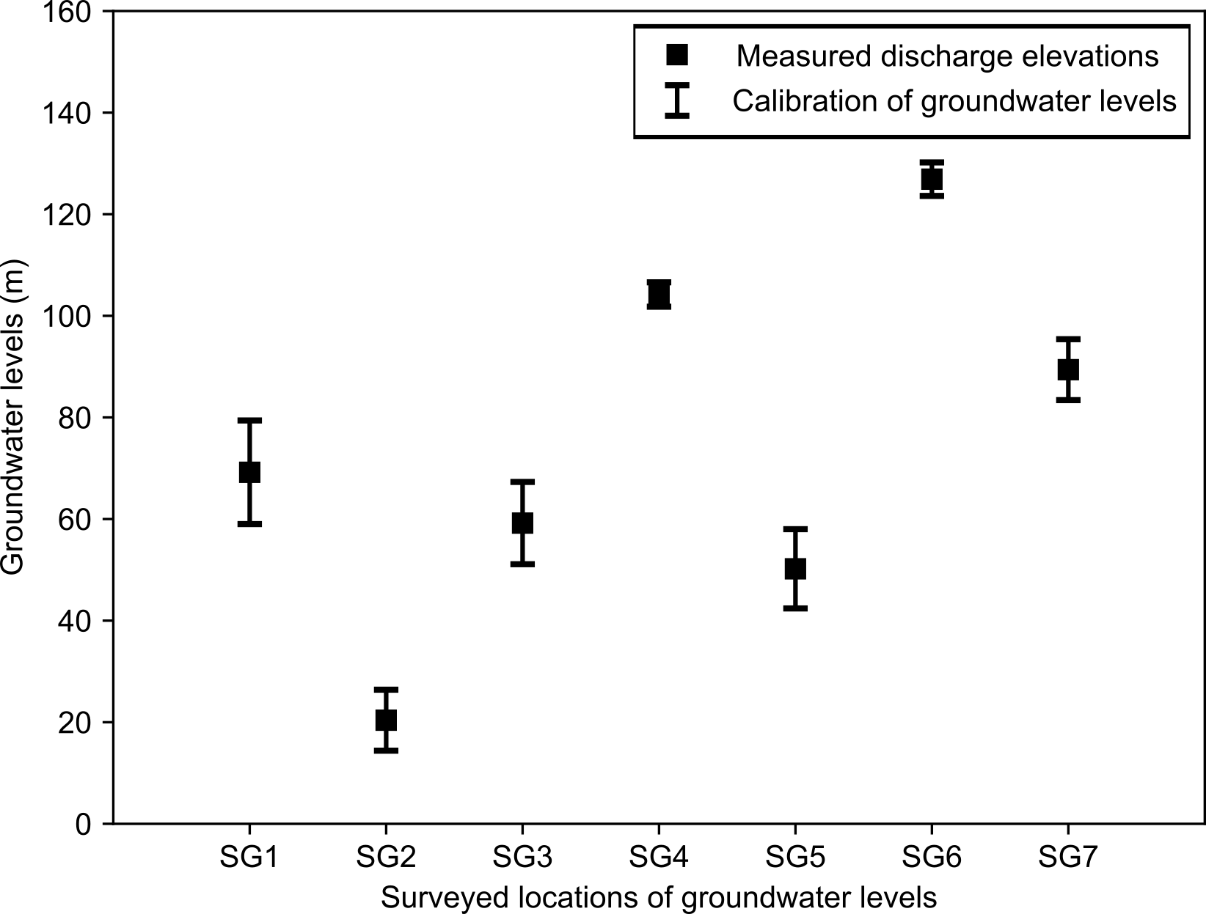


Figure S7. Calibration of the model parameters from the surveyed spatial distribution of groundwater discharge elevations and calculated groundwater levels at the mountainous areas. The locations of the measured groundwater discharge elevations are presented in Fig. S2(a). Bars show 3-standard-deviation uncertainties and were obtained through bootstrapping.


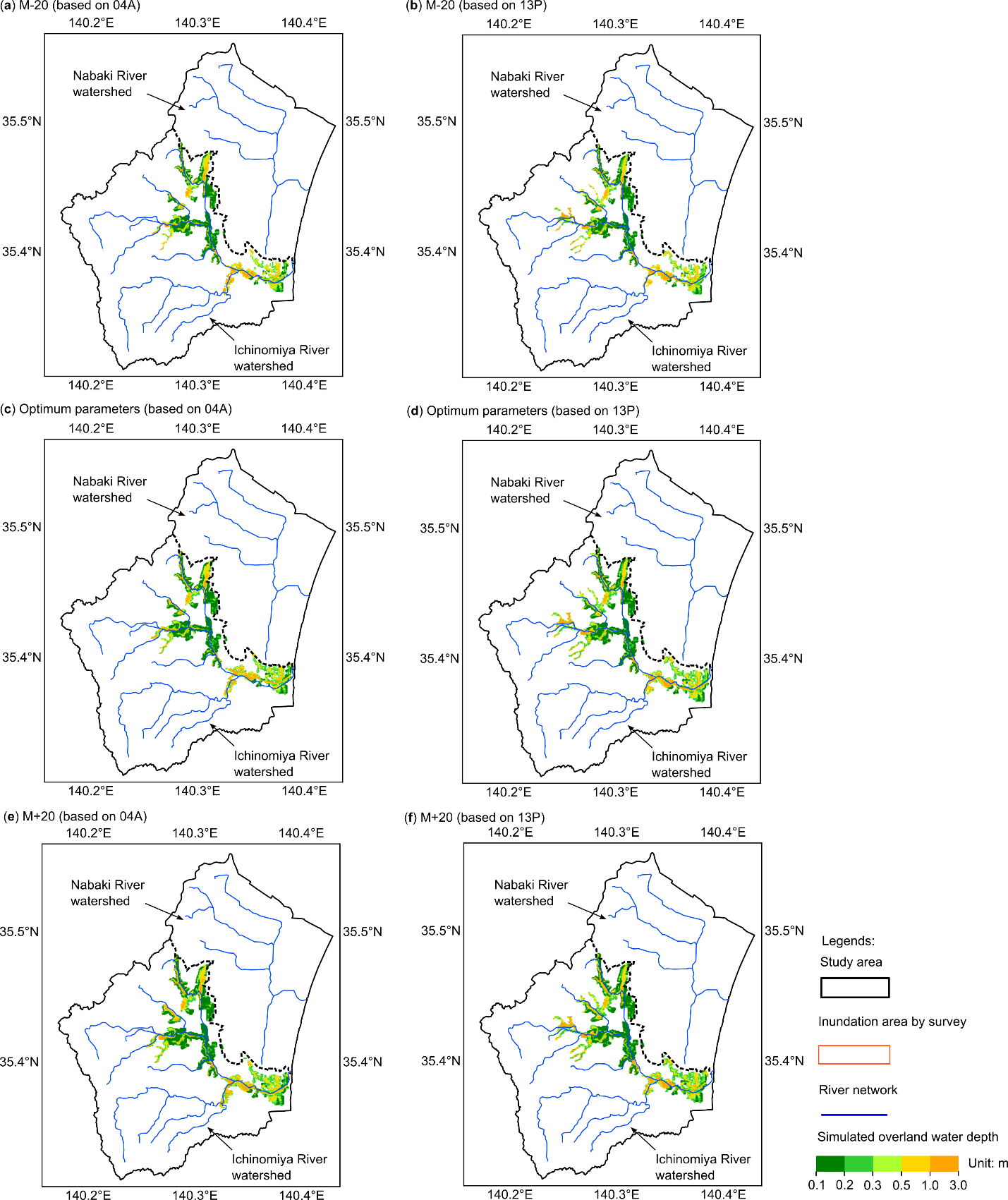


Figure S8. Calculated spatial distributions of inundation areas by changing the Manning’s roughness coefficient. (Figure generated using Matplotlib v. 2.2.3, [www.matplotlib.org](http://www.matplotlib.org/).)


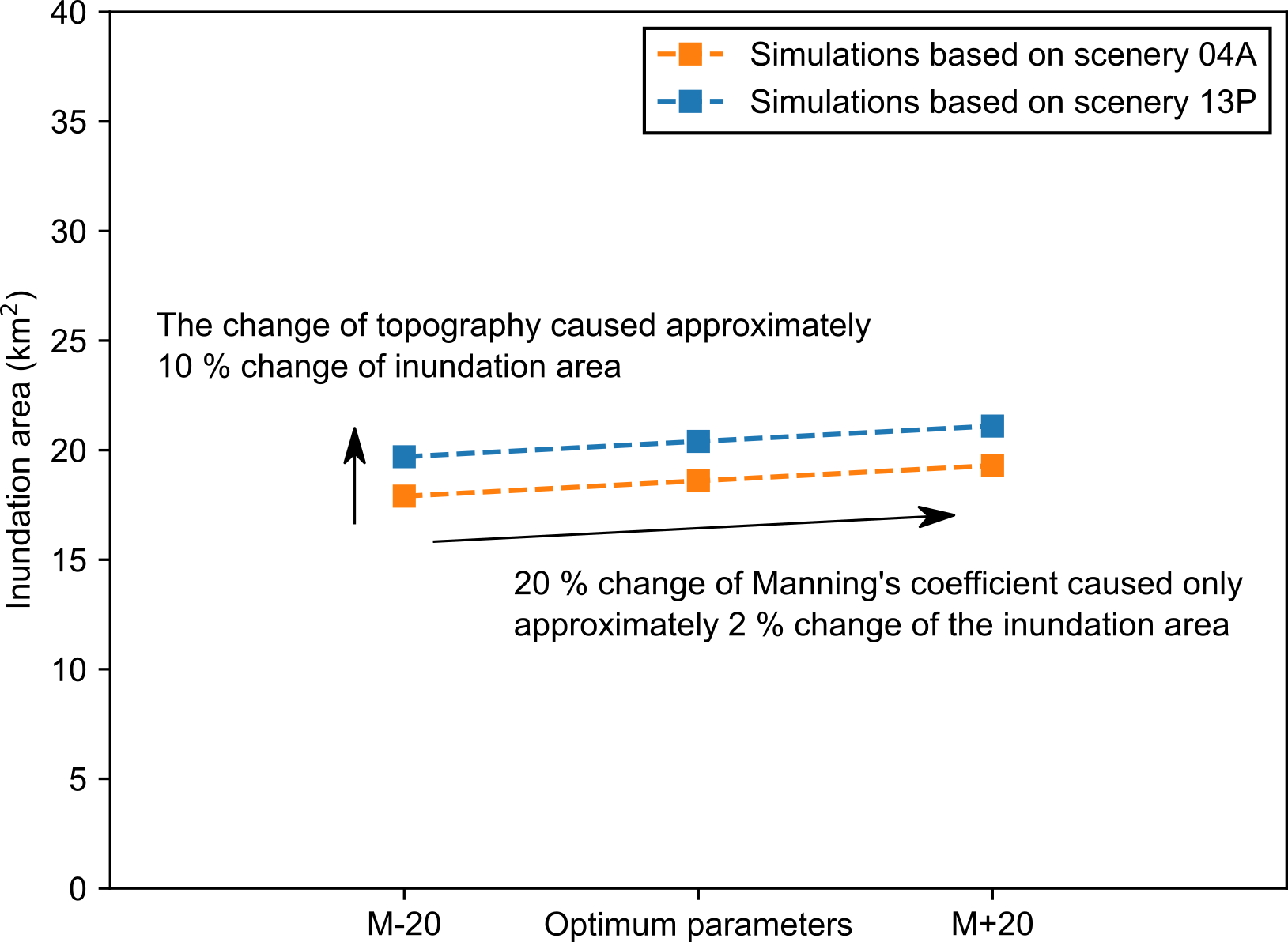


Figure S9. The sensitivity of Manning’s roughness coefficient to the simulated inundation areas.


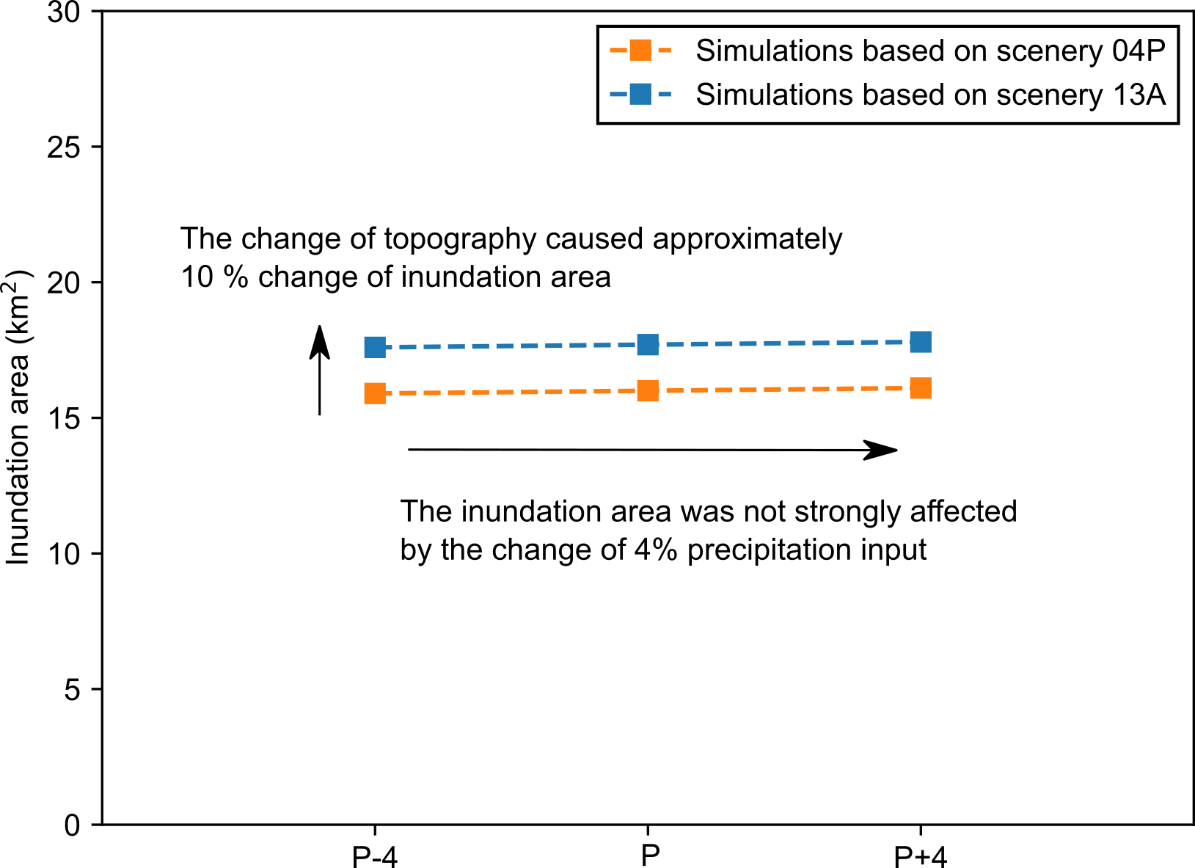


Figure S10. The sensitivity of input precipitation to the simulated inundation areas.
